# Recombinant expression of thermostable processive *Mt*EG5 endoglucanase and its synergism with *Mt*LPMO from *Myceliophthora thermophila* during the hydrolysis of lignocellulosic substrates

**DOI:** 10.1186/s13068-017-0813-1

**Published:** 2017-05-15

**Authors:** Anthi Karnaouri, Madhu Nair Muraleedharan, Maria Dimarogona, Evangelos Topakas, Ulrika Rova, Mats Sandgren, Paul Christakopoulos

**Affiliations:** 1 Biochemical Process Engineering, Chemical Engineering, Department of Civil, Environmental and Natural Resources Engineering, Luleå University of Technology, Luleå, Sweden; 2 Biotechnology Laboratory, Department of Synthesis and Development of Industrial Processes, School of Chemical Engineering, National Technical University of Athens, Athens, Greece; 3 Department of Chemistry and Biotechnology, Swedish University of Agricultural Sciences, Uppsala, Sweden

**Keywords:** *Myceliophthora thermophila*, Endoglucanase, *Pichia pastoris*, Processivity, LPMO, Viscosity

## Abstract

**Background:**

Filamentous fungi are among the most powerful cellulolytic organisms in terrestrial ecosystems. To perform the degradation of lignocellulosic substrates, these microorganisms employ both hydrolytic and oxidative mechanisms that involve the secretion and synergism of a wide variety of enzymes. Interactions between these enzymes occur on the level of saccharification, i.e., the release of neutral and oxidized products, but sometimes also reflected in the substrate liquefaction. Although the synergism regarding the yield of neutral sugars has been extensively studied, further studies should focus on the oxidized sugars, as well as the effect of enzyme combinations on the viscosity properties of the substrates.

**Results:**

In the present study, the heterologous expression of an endoglucanase (EG) and its combined activity together with a lytic polysaccharide monooxygenase (LPMO), both from the thermophilic fungus *Myceliophthora thermophila*, are described. The EG gene, belonging to the glycoside hydrolase family 5, was functionally expressed in the methylotrophic yeast *Pichia pastoris*. The produced *Mt*EG5A (75 kDa) featured remarkable thermal stability and showed high specific activity on microcrystalline cellulose compared to CMC, which is indicative of its processivity properties. The enzyme was capable of releasing high amounts of cellobiose from wheat straw, birch, and spruce biomass. Addition of *Mt*LPMO9 together with *Mt*EG5A showed enhanced enzymatic hydrolysis yields against regenerated amorphous cellulose (PASC) by improving the release not only of the neutral but also of the oxidized sugars. Assessment of activity of *Mt*EG5A on the reduction of viscosity of PASC and pretreated wheat straw using dynamic viscosity measurements revealed that the enzyme is able to perform liquefaction of the model substrate and the natural lignocellulosic material, while when added together with *Mt*LPMO9, no further synergistic effect was observed.

**Conclusions:**

The endoglucanase *Mt*EG5A from the thermophilic fungus *M. thermophila* exhibited excellent properties that render it a suitable candidate for use in biotechnological applications. Its strong synergism with LPMO was reflected in sugars release, but not in substrate viscosity reduction. Based on the level of oxidative sugar formation, this is the first indication of synergy between LPMO and EG reported.

**Electronic supplementary material:**

The online version of this article (doi:10.1186/s13068-017-0813-1) contains supplementary material, which is available to authorized users.

## Background

Cellulolytic enzymes are portrayed as the key factors implied in the natural biodegradation processes in which plant materials are efficiently degraded by cellulolytic protozoa, bacteria, actinomycetes, and fungi [1]. Filamentous fungi are extensively studied candidates for decomposition of organic matter and, more specifically of lignocellulosic substrates [2]. *Myceliophthora thermophila*, a filamentous fungus that belongs to the phylum *Ascomycota*, features an exceptionally powerful cellulolytic system for the degradation of biomass [3]. The system of *M. thermophila* consists of a repertoire of enzymes with endoglucanase (EG), cellobiohydrolase (CBH), and β-glycosidase (BGL) activity [4]. Throughout the genome of this fungus, there are eight nucleotide sequences encoding EG activity, distributed to glycoside hydrolase (GH) families 5, 7, 12, 45, all predicted to be secreted and possess several O- and N-glycosylation sites [5]. Four enzymes (EG60, EG51, EG28, and EG25 belonging to families GH5, 7, 12, and 45, respectively) have been isolated from the culture broth of the C1 mutant strain of *M. thermophila* [6, 7], while one EG from family GH5 (*St*Cel5A) has been heterologously expressed in *Aspergillus niger* [8]. Another EG from family GH7 (*Mt*EG7a), expressed in *Pichia pastoris,* represents one of the most thermostable fungal enzymes reported up to date, with high activity on substrates containing β-1,4-glycosidic bonds [9]. EGs typically degrade cellulose chain by randomly attacking internal sites, thereby creating new chain ends and liquefying rapidly and efficiently high-solids lignocellulosic substrates [9]. Fungal EGs belonging to family GH5, hydrolytically cleave the glycosidic linkages of their substrates using the double-displacement retaining mechanism [10]. It has been shown that some of them possess active site tunnels capable of simultaneous substrate binding and catalysis, so the enzyme has the ability to catalyze consecutive sugar cleavage without releasing the substrate during its passage [11, 12]. This characteristic, called *processivity*, improves the catalytic efficiency for hydrolysis of crystalline substrates and is mainly attributed to CBHs [13]; however, there have been reported EGs with processive activity, which release soluble oligosaccharides before detaching from the substrate [14]. Apart from their implications for biofuel production, EGs have been used together with other cellulases and hemicellulases in numerous industrial applications, involving their use in fiber modification [15], reduction of the viscosity of pulps and juices in food industry, as well as processing of non-starch compounds in food technology [16]. Until recently, only hydrolytic enzymes, like EGs and CBHs, were thought to play a role in the degradation of recalcitrant cellulose and hemicelluloses to fermentable sugars. Recent studies demonstrate that enzymes from the auxiliary activities (AAs), especially the recently discovered AA9 family (previously GH family 61), show lytic polysaccharide monooxygenase activity (LPMO) and have a substantial effect when combined with common cellulases [17, 18]. LPMOs are metalloenzymes that have been shown to enhance the hydrolytic potential of a cellulase mixture during the enzymatic hydrolysis of lignocellulosic substrates [19]. LPMOs’ oxidative mechanism to cleave cellulose is thought to be mediated through a divalent metal ion coordinated by a histidine-brace. The three nitrogen ligands of the latter are provided by the amino group and side chain of an N-terminal His residue and the side chain of a second His residue. LPMO active site is planar, in contrast to cellulolytic hydrolases that possess a channel or cleft shape active site and cleave cellulose by a mechanism that involves the conserved carboxylic acid residues located within this site [20–22]. Although a generalized model mechanism of LPMOs’ action has not yet been verified, the oxidation at the C1 and/or C4 carbon in the glucose ring structure [23] and the need of a reductant cofactor are highlighted as the most represented common features of these enzymes [21, 24]. This cofactor works as an external electron donor to enhance LPMO’s activity and can be lignin [23]. Cannella et al. [25] showed that around 4.1% of the glycosidic bonds in cellulose were oxidatively cleaved by LPMO enzymes, thus facilitating the access of hydrolytic enzymes to the substrate. LPMOs have been suggested to promote the amorphogenesis of the substrate, thus enhancing the effectiveness of cellulase enzymes [26]. *Tt*PMO1 isolated from *Thielavia terrestris* [27] and *Mt*LPMO9 from *M. thermophila* [28, 29] have been shown to considerably enhance the glucose release from cellulosic substrates when added to a cellulase cocktail. A direct correlation between saccharification yields and LPMO activity levels of commercial enzyme cocktails has been reported, indicating the key role of LPMOs in biomass degradation [30]. Strong synergistic effects of LPMOs with EG I and CBH I reported in the literature are indicative of the cooperative interactions of the monooxygenases with specific cellulolytic activities [28, 31].

In this study, the cloning, heterologous expression, and characterization of a GH5 EG from *M. thermophila* are described. The corresponding gene was isolated from the fungal genomic DNA, then cloned and amplified in *E. coli* strains and finally heterologously expressed in *P. pastoris*. The recombinant protein was secreted to the culture medium, purified to homogeneity, and characterized. The productivity of *P. pastoris* in shake flasks is typically low and is improved greatly when the expression was carried out in bioreactor. The controlled environment of the bioreactor resulted in an efficient growth of the organism to high cell densities. Moreover, *P. pastoris* cells grown in bioreactor are fed with methanol at growth-limiting rates; this state has been reported to trigger the transcription and, subsequently, the expression initiated by the AOX1 promoter in higher levels compared to batch cultures where cells grow in excess of methanol [32]. Therefore, improving the fermentation methodology is essential for *P. pastoris*-based processes, including substrate feeding strategies, oxygen supplementation to allow higher cell densities while avoiding oxygen limitation, and mixed-substrate feeding strategies [33–35]. In this study, two basic strategies were followed for EG production. These strategies include control of proteolysis through low temperature and addition of amino acid-rich supplements to the culture medium, as well as smooth transition of cell culture from glycerol to methanol feed phase. This procedure resulted in the successful production of the recombinant protein in the culture medium at high levels. Characterization of the enzyme properties, as well as the mode of action in concert with *Mt*LPMO9 from *M. thermophila* is also described.

## Methods

### Enzymes and chemicals

KOD Hot Start^®^ DNA polymerase was obtained from Novagen (USA) and restriction enzymes were obtained from TAKARA (Japan). Nucleospin Gel Clean-up and GeneJET Plasmid Miniprep kits were purchased from Macherey–Nagel (Germany) and Fermentas (USA), respectively. Carboxymethyl cellulose (CMC) and cello-oligosaccharides (DP 2–5) were obtained from Sigma-Aldrich (St. Louis, MO, USA). Cellohexaose and barley β-glucan were purchased from Megazyme, while microcrystalline cellulose Avicel was from Merck (Darmstadt, Germany). All other chemicals used in this study were of analytical grade. Phosphoric acid swollen cellulose (PASC) was prepared from Avicel, following the protocol initially described by Wood [36]. In order to estimate the conversion of crystalline to amorphous cellulose, a PANalytical Empyrean X-ray diffractometer, equipped with a graphite monochromator and a PIXcel3D detector, was used to determine the X-ray diffraction (XRD) patterns of Avicel and PASC. CuKα radiation with *λ* = 1.540598 at 45 kV and 40 mA was used in the 2*θ* range 5–90^ο^ at a scanning speed of 0.026°/s. Hydrothermal pretreatment of wheat straw (*Triticum aestivum* L.) was conducted using a microwave digestion equipment, at 195 °C for 15 min, as previously described for the pretreatment of sweet sorghum bagasse [37]. Organosolv-pretreated birch and spruce (182 °C for 1 h, 1% H_2_SO_4_, 60% EtOH) [38] were used for the hydrolysis experiments.

### Strains, growth conditions, and DNA extraction

*Myceliophthora thermophila* ATCC 42464 stock cultures were maintained on agar slants containing 1.5% malt-peptone, at 4 °C. *P. pastoris* cultivation was performed in shake flasks at 30 °C, following the instructions in the EasySelect™ *Pichia Expression Kit* (Invitrogen, USA). Genomic DNA from *M. thermophila* was prepared and isolated according to the procedure described previously [39]. For the cloning of the EG and LPMO genes from *M. thermophila*, *Escherichia coli* One Shot^®^ Top10 (Invitrogen, USA) and Zero Blunt^®^ PCR Cloning Kit (Invitrogen, USA) were used as the host–vector system. For the protein expression, *P. pastoris* host strain X33 and the vector pPICZαC (Invitrogen, USA) were used.

### Cloning of *eg5a*

For the expression and secretion of *Mt*EG5A, an *E. coli*/*P. pastoris* shuttle vector, pPICZαC, was used. The pPICZαC vector contains the *AOX1* promoter that ensures the tightly regulated, methanol-induced protein expression, the *Saccharomyces cerevisiae* α-factor secretion signal located upstream of the multiple cloning sites and a C-terminal polyhistidine tag [40]. The gene coding the hypothetical protein *Mt*EG5A (Model ID 86753, chromosome 1:2823610-2825549) was PCR amplified from genomic DNA using primers **EF/ER** (see Additional file 1: Table S1A) designed according to the available gene sequence (http://genome.jgi-psf.org/, DOE Joint Genome Institute) [4] including the *Cla*I and *Xba*I restriction enzyme sites at their respective 5′-ends. For the PCR, a high-fidelity KOD Hot Start^®^ DNA polymerase that produced blunt ends was used. DNA amplification was carried out with 30 cycles of denaturation (20 s at 95 °C), annealing (10 s at 56 °C), and extension (25 s at 70 °C), followed by 1 min of further extension at 70 °C. The PCR product was cloned into the pCRBlunt^®^ vector, following the instructions described in the Zero Blunt^®^ PCR Cloning Kit.

The *overlap extension polymerase chain reaction* (OEPCR) technique was adopted for the intron 1 and intron 2 removal, using the polymerase KOD Hot Start^®^ [39]. Two complementary DNA primers per intron and two external primers (**EF/Ee1R**, **Ee2F/Ee2R**, **Ee3F/ER;** see Additional file 1: Table S1B) were used to generate two DNA fragments that possess overlapping ends. The recombinant plasmid pCRBlunt/*eg5a*, at an appropriate dilution, was used as template DNA and the PCR conditions for each reaction are given as the following: 95 °C for 2 min, ensued by 30 cycles of 95 °C for 20 s, annealing for 10 s and extension step, with a final extension step at 70 °C for 1 min. Annealing and extension conditions for each fragment are described in Additional file 1: Table S1B. The two PCR products were combined together into a “*fusion*” fragment and amplified further by overlapping PCR due to their complementary fractions, through the utilization of the two external primers, **EF** end **ER**. Overlapping PCR was performed with an initial denaturation step at 95 °C for 2 min, followed by 45 cycles at 95 °C for 20 s, 56 °C for 10 s, 59 °C for 26 s, and a final extension step at 70 °C for 1 min. The produced *eg5a* DNA was digested with the restriction enzymes *Cla*I and *Xba*I, gel-purified, and subsequently cloned into the pPICZαC vector. The resulting pPICZαC/*eg5a* was used for the transformation of *E. coli* TOP10F’cells. After confirmation of gene insertion with restriction analysis, the recombinant vector was linearized with *Sac*I and transformed into *P. pastoris* by electroporation.

The transformation of pPICZαC/*eg5a* into *P. pastoris* and cultivation in shake flasks were performed following the protocols described in the EasySelect Pichia Expression Kit (Invitrogen, USA). The tranformants were isolated on plates containing Zeocin™ at a concentration of 100 μg/mL.

In order to demonstrate the clone that exhibited the highest protein expression levels, different *P. pastoris* single colonies were cultivated in glycerol-containing medium for 18–24 h, at 30 °C in a shaker (200 rpm) and then inoculated into methanol-containing medium (0.5% v/v) for induction of the expression. The protein secreted in the culture supernatant was tested for endoglucanase activity against β-glucan 1% w/v in 100 mM phosphate–citrate buffer pH 5.0, after 24 h of incubation at 30 °C and 200 rpm. The clone with maximal activity was chosen for enzyme production and further characterization studies. Enzyme production was performed at 30 °C for 6 days (200 rpm) with the daily addition of 0.5% v/v methanol for the induction.

### Production and purification of recombinant *Mt*EG5A

High cell-density cultivation of *P. pastoris* cells in bioreactor was performed in the basal salt medium (BSM), following the *Pichia* Fermentation Process Guidelines by Invitrogen, as described previously [41]. *P. pastoris* cells were cultivated overnight in glycerol-containing medium that corresponded to 5–10% of the initial bioreactor volume, at 30 °C under agitation (200 rpm). Then, the culture was inoculated to bioreactor BSM medium containing 3% glycerol and the temperature, agitation, and aeration operating conditions were adjusted to 28 °C, 800 rpm, and 4 vvm, respectively. 4.35 mL of PTM_1_ trace salts per liter of the cultivation medium was added aseptically and pH was adjusted to 5.0 with 28% ammonium hydroxide.

The glycerol batch cultivation in glycerol medium lasted for ~24 h and was succeeded by a 5-h step of fed-batch glycerol one; during this step, 50% w/v glycerol, with PTM_1_ salts (12 mL/lt glycerol) was fed at an initial flow rate of 12 mL/h/lt of culture medium and was gradually reduced until it was fully consumed. Temperature was now set to 23 °C and small amount of methanol was added manually with syringe. After glycerol had been consumed (as indicated by a sharp increase in the dissolved oxygen (DO) tension, a casamino acids solution was added (3 g/lt medium) and then, a feed of 100% MeOH, with PTM_1_ salts (12 mL/lt MeOH) was initiated at a flow rate of 1.9 mL/h/lt. During the methanol fed-batch phase, the methanol feed rate was increased manually, after monitoring the methanol consumption rate indirectly by stopping the feed and checking the “*lag time*.” After 8 h, feed rate was set to 5.46 mL/h/lt and maintained for ~20 h. Then, the temperature was reduced to 21 °C and pure oxygen supply was set to maintain DO levels between 15 and 20%. Induction time lasted approximately 145–170 h in total. Samples were taken and analyzed for the determination of cell growth (OD_600_ and wet cell weight), protein concentration, and endoglucanase activity.

For the purification of the recombinant enzyme, the culture supernatant was centrifuged, concentrated 30-fold using a LabScale Tangential flow filtration system (TFF with membrane Pellicon XL Ultrafiltration Module Biomax, exclusion size 10 kDa; Millipore, Billerica, USA), and dialyzed for 8–12 h at 4 °C against a 20 mM Tris–HCl/300 mM NaCl buffer (pH 8.0). *Mt*EG5A was then purified by single-step immobilized metal ion affinity chromatography (IMAC) on an ÄKTA Prime Plus system equipped with a cobalt-charged resin, at a flow rate of 2 mL/min, using 0–100 mM imidazole gradient. Fractions (2 mL) containing EG activity were concentrated and were checked by sodium dodecyl sulfate-polyacrylamide gel electrophoresis (SDS-PAGE) using 12% acrylamide-separating gels. *Mt*EG5A was further polished using S300 Gel Filtration chromatography column to remove trace EG contaminants and eluted in 100 mM citrate–phosphate buffer pH 5.0.

### Enzyme assay

*Mt*EG5A activity was determined on β-glucan 0.1% w/v for 15 min, at 60 °C in 100 mM citrate–phosphate buffer pH 5.0. The concentration of reducing ends was determined using the dinitrosalicylic acid reagent (DNS) [42] and glucose for the standard curve. One unit (U) of EG activity was defined as the amount of enzyme that liberated 1 μmol of glucose equivalents per minute under assay conditions. The amount of protein was estimated by the BCA protein assay microplate procedure (Pierce Chemical Co., Rockford, IL), following the manufacturer’s recommendations, using bovine serum albumin as standard [43].

### Enzyme characterization

The optimal temperature of *Mt*EG5A was estimated by using the standard assay procedure at temperatures varying from 30 to 90 °C. To determine the temperature stability, 0.23 mg of purified *Mt*EG5A was incubated at various temperatures for different time intervals, and the residual activity was measured under the standard assay procedure. The optimal pH was determined by the standard assay at 60 °C over the pH range 3.0–11.0 using either 0.1 M citrate–phosphate buffer pH 3.0–7.0, 0.1 M Tris–HCl pH 7–9, or 0.1 M glycine–NaOH buffer pH 9–11. The stability at different pH conditions was determined after incubating *Mt*EG5A in the above buffers at 4 °C for 24 h and measuring the remaining activity under the standard assay.

To investigate substrate specificity of *Mt*EG5A, different substrates (CMC, Avicel, and PASC) were selected. Enzyme activity was determined after incubation in 0.1 M citrate–phosphate buffer (pH 5.0) containing 1.0% of each substrate at 60 °C for 15 min (CMC) or 60 min (Avicel and PASC). The amount of reducing sugars produced was estimated using the DNS method, as described above. For studying the hydrolysis products of cello-oligosaccharides under the action of the purified enzyme, 90 ng of *Mt*EG5A and 40 μM cellotriose (G3), cellotetraose (G4), cellopentaose (G5), or cellohexaose (C6), respectively, in 50 mL citrate–phosphate buffer (pH 5.0) were incubated at 50 °C for 100 min. Fucose was used as a reaction internal standard at a final concentration of 20 μM. During hydrolysis, samples of 250 μL were taken and inactivated by boiling for 15 min prior to analysis using high-performance anion exchange chromatography (HPAEC). The analysis was conducted using an ICS 5000SP system (Dionex, Thermo Fisher Scientific Inc.) with a pulsed amperometric detector equipped with a disposable electrochemical gold electrode, using a CarboPac PA1 4 × 250 mm analytical column and a CarboPac PA1 4 × 50 mm guard column, at 30 °C. 10 μL of samples were injected and the reaction products were eluted at 1 mL/min with initial conditions set to 0.1 M NaOH (100% eluent A). This step was succeeded by a linear gradient toward 10% eluent B (1 M NaOAc in 0.1 M NaOH) after 10 min and 30% B after 25 min; a 5 min exponential gradient (Dionex curve 6) to 100% B followed. Between separate injections, a 10 min stabilization step (100% A) was interjected. Integration of chromatograms was performed using Chromeleon 7.0 software. For the identification and quantification of products released from hydrolysis, d-glucose and G2–G6 cello-oligosaccharides were used as carbohydrate standards. The first-order reaction was used to describe the enzymatic hydrolysis of cello-oligosaccharides, with the assumption that the condition [E_0_] ≪ [S_0_] ≪ *K*_*m*_ is satisfied (where [E_0_] and [S_0_] stand for the concentrations of the enzyme and substrate, respectively). The estimation of the catalytic efficiency of *Mt*EG5A against cello-oligosaccharides was made using the equation *k* × *t* = ln([S_0_]/[S_*t*_]) that represents the integrated rate equation for the first-order kinetics, where *k* = (*k*_cat_/*K*_m_) [enzyme], whereas [S_0_] and [S_*t*_] correspond to substrate concentration when the reaction starts and at a specified time during the reaction, respectively [44].

Processivity of the enzyme was determined following the soluble-to-insoluble reducing sugar ratio assay [45]. *Mt*EG5A was incubated with 50 mg filter paper Whatman No. 1 in 0.1 M citrate–phosphate buffer (pH 5.0), at 60 °C for 1 h, under agitation (700 rpm). The remaining insoluble substrate was separated from the reaction supernatant by centrifuge, and the amount of reducing sugars in both soluble and insoluble fractions was determined by DNS method, as previously described [46]. The remaining filter paper strips were washed with buffer prior to sugar determination. Processivity was also estimated by the ratio of G2 to G3 that was released from PASC and determined with HPAEC, as described above. *Mt*EG5A was incubated with PASC 1.5% (w/v) in 50 mM citrate–phosphate buffer (pH 5.0), at a loading of 10 mg/g substrate, at 50 °C, and samples were taken for analysis after 2, 4, 8, and 24 h. The GH family 7 EG *Mt*EG7A from *M. thermophila* [9] was also assayed under the same conditions in order to compare the different modes of activity. The combined effect of *Mt*EG5A together with *Mt*EG7A was also evaluated on PASC 1.5% (w/v), at 50 °C, following the same procedure as described above. The activity of *Mt*EG5A toward wheat straw, birch, and spruce was evaluated at an initial dry matter (DM) of 3% (w/v) in 50 mM citrate–phosphate buffer (pH 5.0), at a loading of 10 mg/g substrate, at 50 °C, and samples were taken after 24 h for analysis with HPAEC, as described above.

### Cloning of *lpmo9*

*Mt*LPMO9 (XP_003661787) from *M. thermophila* was cloned and expressed to study its combined effect together with *Mt*EG5A. The vector pPICZαC and *P. pastoris* host strain X33 were used for the expression and secretion of *Mt*LPMO9, similar to *Mt*EG7A. In order to achieve enzyme production with the authentic N-terminus, i.e., with the copper-coordinating histidine at position 1, the native signal peptide was employed for the secretion of recombinant *Mt*LPMO9. To remove the *S. cerevisiae* α-factor secretion signal, the pPICZαC vector was digested with restriction enzymes *Bst*BI and *Xba*I. The gene coding the hypothetical protein *Mt*LPMO9 including the signal peptide sequence, was PCR amplified from genomic DNA using primers **LF/LR** (see Additional file 1: Table S3) designed according to the available gene sequence (http://genome.jgi-psf.org/, DOE Joint Genome Institute) [4] including the *Bst*BI and *Xba*I restriction enzyme sites at their respective 5′-ends. PCR was performed using KOD Hot Start^®^ DNA polymerase, under the following conditions: 30 cycles of denaturation (20 s at 95 °C), annealing (10 s at 56 °C), and extension (21 s at 70 °C), followed by 1 min of further extension at 70 °C. The PCR product was cloned into the pCRBlunt^®^ vector, following the instructions described in the Zero Blunt^®^ PCR Cloning Kit. The expression and purification of *Mt*LPMO9 were performed following the procedure previously described [47].

### Combined activity of *Mt*EG5A with *Mt*LPMO9

To assess their combined effect, *Mt*EG5A was incubated with *Mt*LPMO9 at different enzyme combinations and loadings, at 50 °C, using PASC 1.5% (w/v) in 50 mM citrate–phosphate buffer (pH 5.0) as substrate. Samples were taken for analysis after 30 and 60 min and determination of the soluble products was conducted by HPAEC, as described above.

The ability of *Mt*EG5A to drop the viscosity of PASC and PWS was monitored with dynamic viscosity measurements. The experiments were carried out in a microprocessor-controlled rotational viscometer (Rapid Visco Analyzer, model RVA-4500 Perten Instruments GmbH, Germany) with Thermocline software (TCW version 3.15; Perten Instruments of Australia). All reactions were performed at 55 °C. Liquefaction of pretreated wheat straw (PWS) was monitored in 30 g of 10% w/w DM wheat straw suspension prepared in 50 mM citrate–phosphate buffer (pH 5.0), while PASC was used at a concentration of 1.5% (w/v) in the same buffer (pH 5.0) and a final reaction volume of 18 mL. The reaction mixture was prepared directly into the instrument container and was incubated until the set temperature had been reached. Then, the reaction was started by the addition of 100 mg enzyme/g dry substrate. During the test, the suspension was mixed by a plastic paddle at a constant rate (50 and 100 rpm in case of PASC and PWS, respectively) and the apparent viscosities (cP; 1 cP = 1 mPa s) were recorded at intervals of 4 s for 1 h. Combined activity of *Mt*EG5A and *Mt*LPMO9 was also assessed by adding simultaneously both enzymes in relative proportions of 1:1, at a total enzyme loading of 200 mg/g of substrate.

## Results

### Identification, cloning, and expression of *Mt*EG5A

**Fig. 1:**
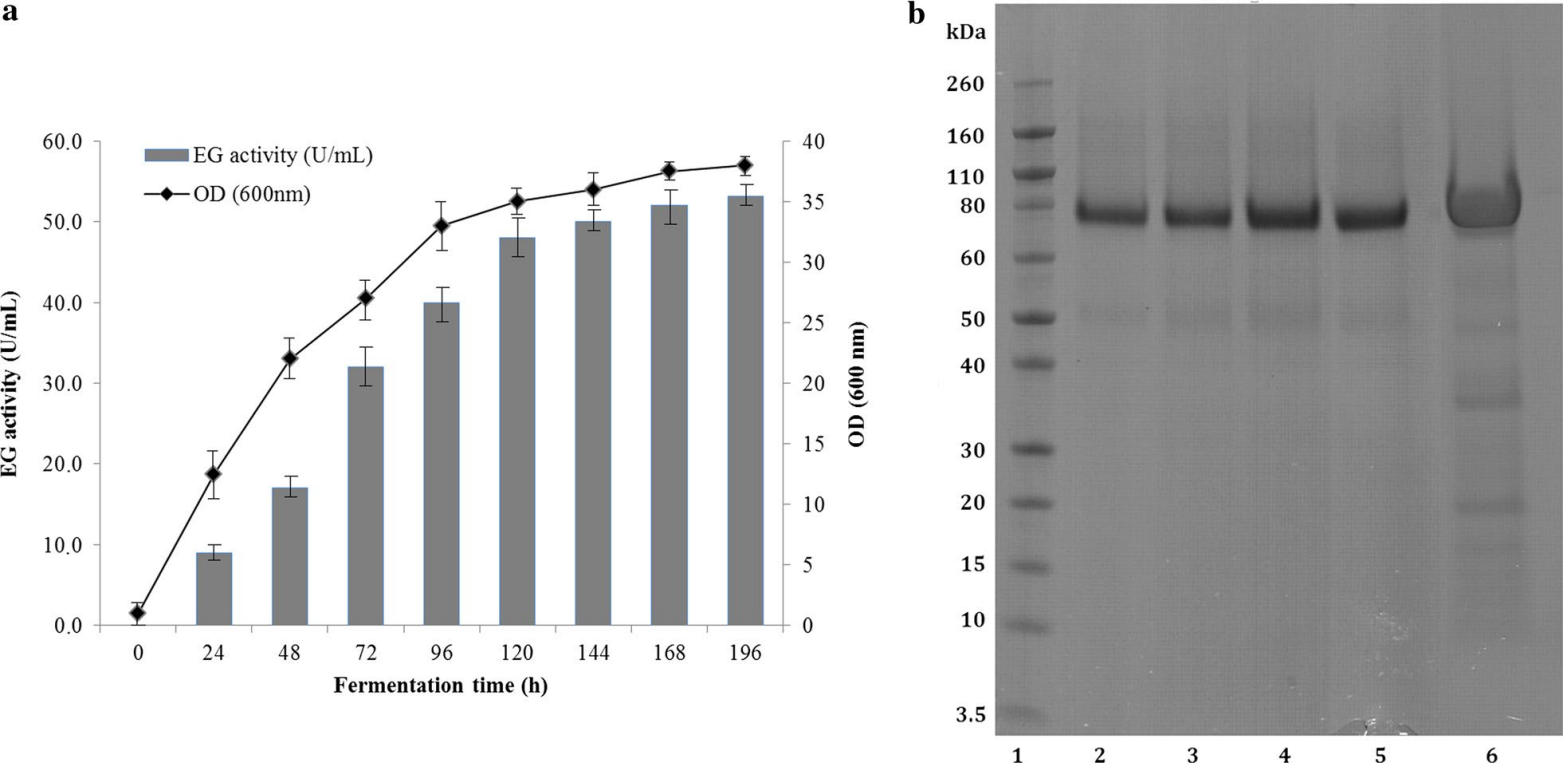
**a** Time course of *Mt*EG5A endoglucanase activity (*gray* bar) and biomass (*black* circle) production of the recombinant *P. pastoris* harboring the *eg5a* gene. The endoglucanase was expressed in culture broth by induction with 0.5% methanol and measured with β-glucan as substrate. **b** SDS-PAGE of *Mt*EG5A during fermentation. *Lane 1* Novex^®^ sharp pre-stained protein marker, *lanes 2*–*5* samples from fermentation culture broth at 99, 118, 130, and 153 h of incubation, *lane 6* culture broth with *Mt*EG5A after ultrafiltration

The genome of *M. thermophila*, the first complete genome described for filamentous fungi [4], contains a vast amount of genes for the hydrolysis of all major polysaccharides that can be found in biomass. The translation of the *eg5a* open reading frame (ORF) (Model ID 86753) from the *M. thermophila* genome database shows significant primary sequence identity with characterized EGs which have been classified to family GH5 on CAZy database (http://www.cazy.org/) [48]. The hypothetical EG showed high sequence identity (65%) with structure solved EG GH5 from *Thermoascus aurantiacus* [GenBank: AFY52522] and 79% with EG from *Penicillium brasilianum* [GenBank: ACB06750]. The putative protein of 86753 was selected as a candidate EG and the corresponding gene, named *eg5a*, was cloned and subsequently used to transform *P. pastoris* X33 under the regulation of the AOX1 promoter. The transformants were selected by their ability to grow on agar plates containing Zeocin™ and to produce crude protein extract with activity against β-glucan, (detailed in “Methods”) in liquid cultures after methanol induction, which is indicative of EG expression (data not shown). All transformants produced a major secreted protein product, named *Mt*EG5A, which appeared on SDS-PAGE as a single band corresponding to ca. 75 kDa, whereas no protein could be detected with the vector control (data not shown). The clone showing the highest activity was isolated for further study. EG activity could be first detected in the medium 24 h after inoculation and increased significantly up after 168 h with a titer of 53 U/ml (Fig. 1a).

### Production of recombinant *Mt*EG5A in bioreactor

**Fig. 2:**
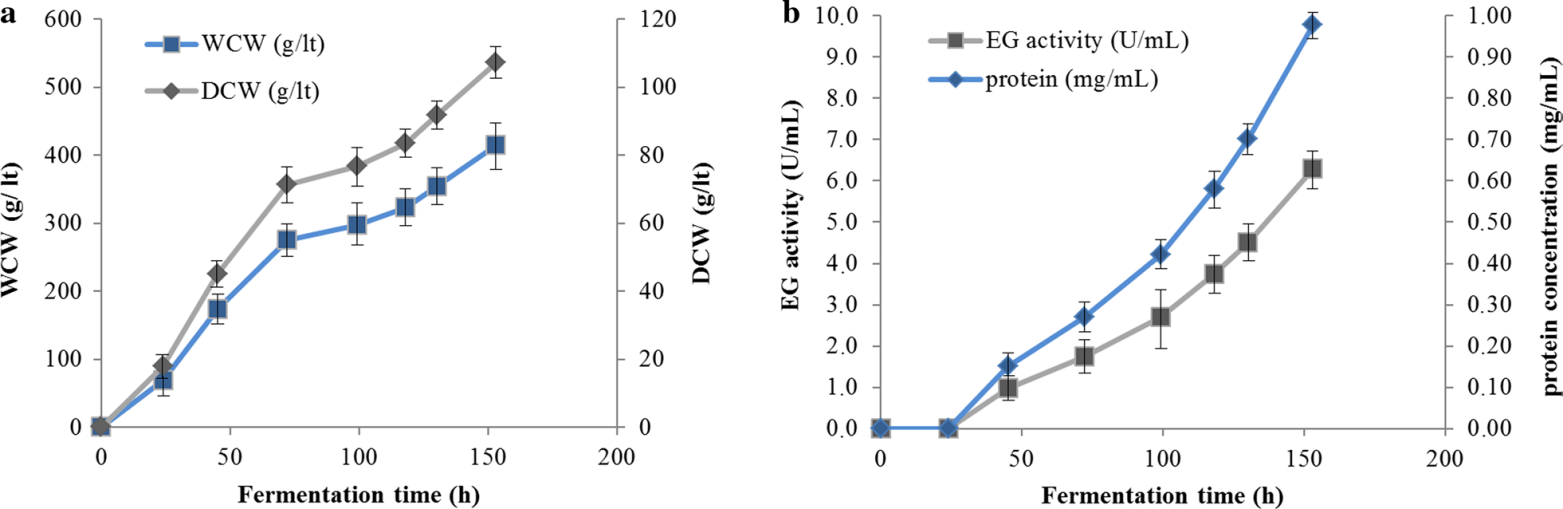
**a** Cell mass concentration during *Mt*EG5A fermentation. Dry cell weight reached 40.1 g/lt after glycerol fed-batch phase and 110.82 g/lt at the end of the cultivation, corresponding to wet cell weight of 183.55 and 421.2 g/lt, respectively. **b** Protein concentration and endoglucanase activity detected in the culture medium during fermentation. At the end of fermentation, the amount of secreted protein reached 0.98 g, corresponding to enzyme activity of 6.3 U/mL. Protein concentration was determined using the BCA protein assay microplate procedure (Pierce Chemical Co., Rockford, IL), according to the manufacturer’s recommendations. Specific activity was tested against β-glucan 0.1% (w/v), pH 5.0, 60 °C in 100 mM phosphate–citrate buffer

After 24 h of batch cultivation in glycerol medium (30 g/lt initial concentration), the dry cell mass of the culture reached about 17.80 g/lt (Fig. 2a). Analysis of the culture supernatant, after growth in glycerol medium, showed absence of recombinant enzyme (based on β-glucan 0.1% w/v activity assay and SDS-PAGE). The glycerol batch was followed by a 5-h step of fed-batch glycerol one, at the end of which the dry cell mass of the cells reached the amount of 40.1 g/lt. Methanol induction time lasted 153 h in total and approximately 700 mL of methanol was consumed. The level of enzyme expression increased with induction time and maximum level obtained was 6.3 U/mL (β-glucan activity for varying time points shown at Fig. 2b). As methanol was used for induction, as well as carbon source, there was an increase in cell-density during the fed-batch phase. At the end of the fermentation, the dry weight of cells reached 110.82 g/lt of culture medium (corresponding to wet cell weight of 421.2 g/lt), while the amount of secreted protein crude produced reached 0.98 g.

### Protein analysis of *Mt*EG5A

The ORF of *eg5a* encodes a protein of 389 amino acids including a secretion signal peptide of 17 amino acids (MKSSILASVFATGAVA) based upon prediction using SignalP v4.0 [49]. The predicted mass and isoelectric point (pI) of the mature protein were 40.85 kDa and pH 5.07, respectively, as calculated using the ProtParam tool of ExPASy [50] (see Additional file 1: Table S2).

The recombinant *Mt*EG5A was purified from the concentrated culture broth by IMAC. Purification of the recombinant enzyme resulted in 564 mg of pure *Mt*EG5A per lt of culture supernatant. *Mt*EG5A homogeneity was examined on a SDS-PAGE gel, where the enzyme was estimated to have a molecular weight of ca. 75 kDa (Fig. 1b). Even after considering that the *myc* epitope and the polyhistidine tag contribute 2.8 kDa to the size of protein, the estimated molecular weight of *Mt*EG5A appeared much higher than the predicted value using the ProtParam tool (40.85 Da). One possible explanation emerges from the presence of Asn-Xaa-Ser/Thr sequons, which are known to be a consensus motif for N-glycosylation post-translational modifications. Indeed, three N-glycosylation sites were predicted by using the NetNGlyc 1.0 server [51] to occur at Asn residues. Moreover, several Ser and Thr residues are located throughout the flexible linker peptide that connects the catalytic domain and the carbohydrate-binding module (CBM) of the protein; these residues tend to be O-glycosylated by the yeast expression system [52]. Indeed, 18 potential O-glycosylation sites (7 Ser and 11 Thr) were identified, as predicted by using the NetOGlyc 3.1 server [53]. Like most secreted cellulolytic enzymes reported in literature [54], *Mt*EG5A features a bimodular structure containing, apart from the active site a recognizable CBM that belongs to the CBM family 1 of the CAZy database. Through this module, the EG adsorbs on the surface of the cellulosic substrate at the initial step of hydrolysis. CBM is characterized by the presence of disulfide bridges formed by conserved cysteines, as well as five aromatic amino acid residues (Tyr433, Trp441, Tyr451, Tyr455, and Trp459) and two glutamines (Gln435 and Gln456), which are necessary for adsorption [55].

### Properties of *Mt*EG5A

**Fig. 3:**
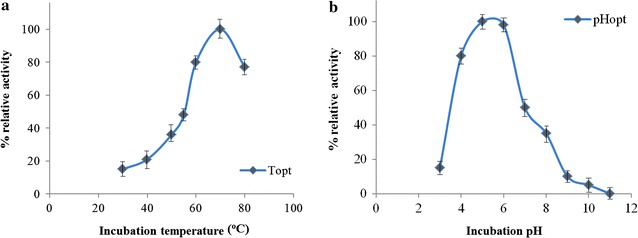
Effect of pH (**a**) and temperature (**b**) on the activity of *Mt*EG5A. The experiment was carried out in duplicates

**Fig. 4:**
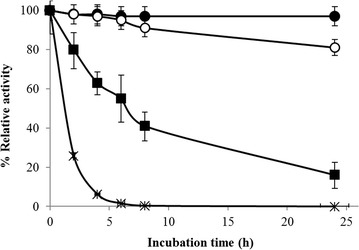
Effect of temperature (*black circle* 50 °C, *white circle* 55 °C, *black square* 60 °C, *black cross* 65 °C) on the stability of *Mt*EG5A. The experiment was carried out in duplicates

The optimum temperature activity of purified recombinant *Mt*EG5A was observed at 70 °C, losing rapidly its activity for temperatures over 80 °C (Fig. 3a). The EG remained fairly stable up to 50 °C after preincubation for 8 h in 100 mM phosphate-citrate buffer (pH 5.0) at different temperatures (Fig. 4). *Mt*EG5A exhibits half-lives of 26.9 and 6.02 h at 55 and 60 °C, respectively. The enzyme presented the highest activity levels at pH 5.0–6.0 (Fig. 3b), while the activity dropped rapidly for pH less than 4 or higher than 7. The enzyme was found remarkably stable in the pH range 3–11 after 24 h retaining 100% of its initial activity.

*Mt*EG5A was assayed for its activity toward different substrates, as described in “Methods”. X-ray diffraction pattern of PASC revealed a partial conversion of crystalline cellulose into amorphous after the addition of 85% phosphoric acid (see Additional file 1: Figure S1), while some areas remained crystalline [56]. The enzyme showed the highest specific activity toward PASC (3.4 U/mg), followed by Avicel (1.63 U/mg), β-glucan (1.19 U/mg), and CMC (0.25 U/mg). In order to investigate the hydrolysis products of cello-oligosaccharides, the hydrolysis patterns of G3, G4, G5, and G6 were analyzed with HPAEC, with the enzyme displaying no detectable activity against cellobiose (G2). The comparison of catalytic efficiency (*k*_cat_ */K*_*m*_) values of *Mt*EG5A against the oligosaccharides showed preference against G6 following a G6 > G5 > G4 > G3 pattern. The *k*_cat_ */K*_*m*_ values against G3, G4, G5, and G6 were 16.4 × 10^4^, 18.9 × 10^5^, 28.2 × 10^5^, and 32.5 × 10^5^ min^−1^ M^−1^, respectively. The hydrolysis of G3 produced G2 and glucose; the hydrolysis of G4 produced only G2; whereas when the enzyme catalyzed the hydrolysis of G5, the products were G2 and G3 which was subsequently cleaved to glucose and G2. In the initial stage of G6 hydrolysis, the reaction products contained G4, G3, G2, and trace amounts of glucose. G4 was further hydrolyzed, while the amounts of G3 and G2 increased constantly as hydrolysis proceeded.

**Fig. 5:**
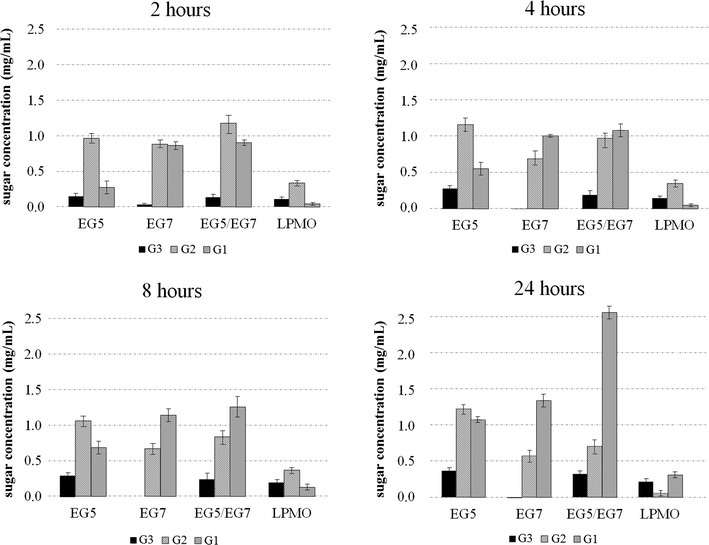
Cello-oligosaccharides (DP 1–3) released by *Mt*EG5A*, Mt*EG7A, and *Mt*LPMO9 on amorphous cellulose PASC after incubation for 2, 4, 8, and 24 h at 50 °C

Processivity of the EG was determined by the soluble-to-insoluble reducing sugar ratio assay and showed that the soluble/insoluble ratio of products released by filter paper hydrolysis under agitation was 3.91 after 1 h of incubation. Estimation of the substrate binding modes and processivity of the enzyme are also verified by the high G2/G3 ratios released from PASC hydrolysis (Fig. 5). G2 was the major reducing sugar released from PASC by *Mt*EG5A, although a low amount of glucose and traces of higher DP oligosaccharides were apparent (see Additional file 1: Figure S2). The concentration of G2 was approximately two fold greater than that of G3. *Mt*EG5A did not cleave G2, but it cleaved larger oligomers quantitatively to a mixture of G2 and glucose [11]. The profiles of *Mt*EG7A hydrolysis products on PASC contained G2, glucose, and a trace of G3 in the initial stage (Fig. 5). The concentration of glucose increased over time and was considerably larger than that of G2. Comparison between *Mt*EG5A and *Mt*EG7A hydrolysis products generated from PASC revealed the differences in catalytic cleavage mode of these two enzymes. Addition of both EGs was shown to have the potential to capitalize on complementary activity to improve cellulose hydrolysis rate and extent. *Mt*EG5A and *Mt*EG7A acted additively on PASC toward the release of glucose after 24 h of incubation (Fig. 5).

**Fig. 6:**
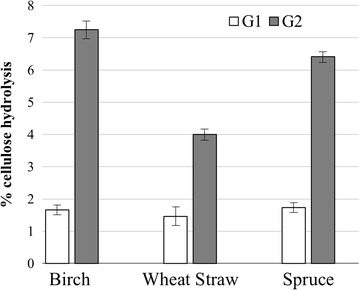
Glucose (G1) and cellobiose (G2) released from organosolv-pretreated spruce, wheat straw, and birch after 24 h of reaction

*Mt*EG5A was able to perform hydrolysis of organosolv-pretreated wheat straw, birch, and spruce (Fig. 6). Analysis of product distribution revealed that the enzyme released mainly cellobiose from all the three substrates tested after 24 h of reaction, in complete agreement with the previous results. Cellobiose yields corresponded to approximately 7% of initial cellulose fraction for birch and spruce and 4% for wheat straw, while glucose yields were lower than 2% for all three substrates.

### Combined activity with *Mt*LPMO9

**Fig. 7:**
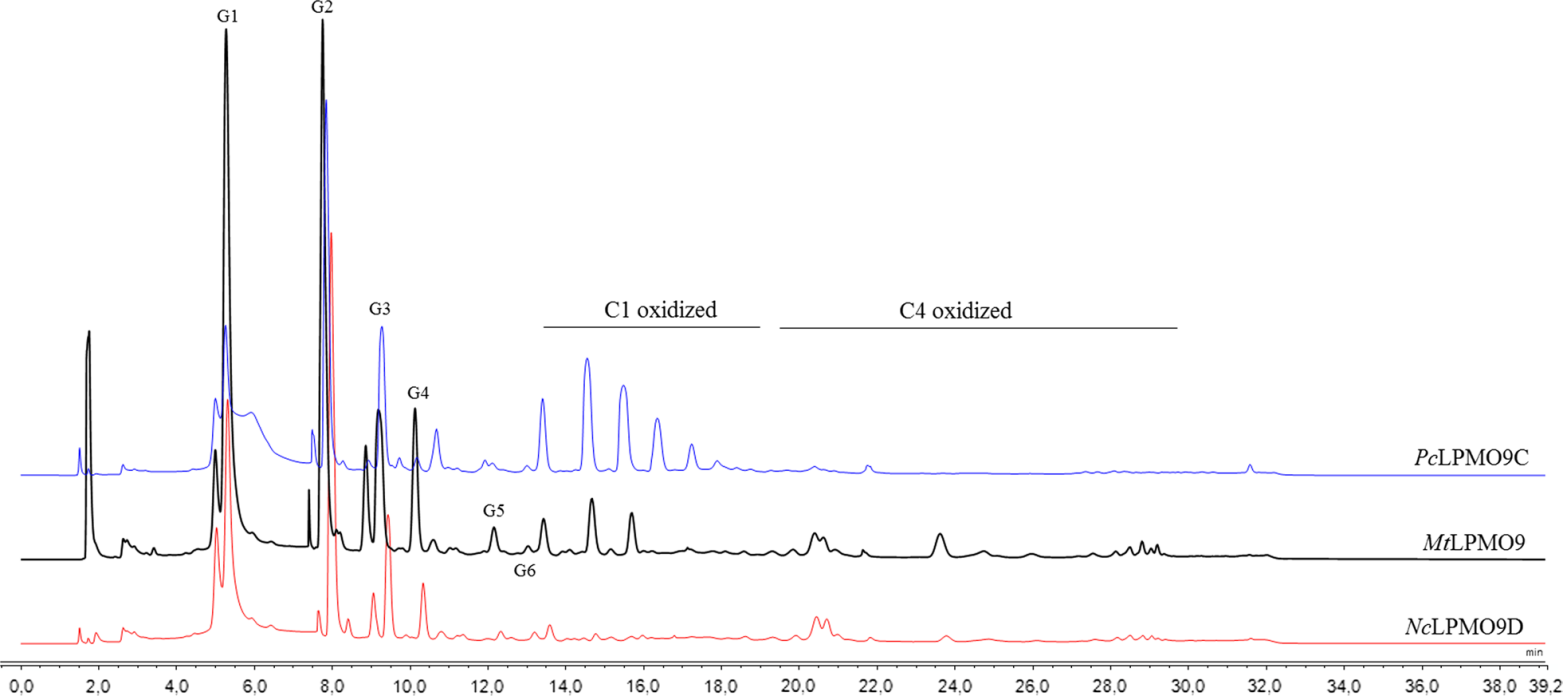
Activity of different LPMOs on PASC. HPAEC-PAD elution patterns of PASC after 24 h incubation with *Mt*LPMO9 (10 mg/g substrate), *Nc*LPMO9C (5 mg/g substrate), and *Pc*LPMO9D (2 mg/g substrate) in the presence of ascorbic acid (10 mM)

Products generated by *Mt*LPMO9 when incubated alone on PASC are depicted in Fig. 5. Glucose and cello-oligosaccharides were released during 24 h of incubation. Higher amounts of G2 were detected in the 1st h of the reaction, while after 24-h incubation, the main non-oxidized product was glucose. Regarding the release of oxidative products, peaks were assigned based on previous assignments [57, 58] and comparison with the activity patterns of *Nc*LPMO9C from *Neurospora crassa* [59] and *Pc*LPMO9D from *Phanerochaete chrysosporium* [60] (Fig. 7). *Nc*LPMO9C is C4-oxidizing LPMO that acts on the C4 atom of sugars leading to a keto-group, while *Pc*LPMO9D oxidizes the C1 atom leading to a lactone and the corresponding aldonic acid. C4-oxidized oligosaccharides elute at 19–30 min and C1-oxidized oligosaccharides elute at 13–19 min retention time in HPAEC-PAD. *Mt*LPMO9 represents an enzyme with dual oxidative regioselectivity producing C1/C4 double-oxidized species that elute in the HPAEC-PAD chromatograms at 13–30 min retention time.

**Fig. 8:**
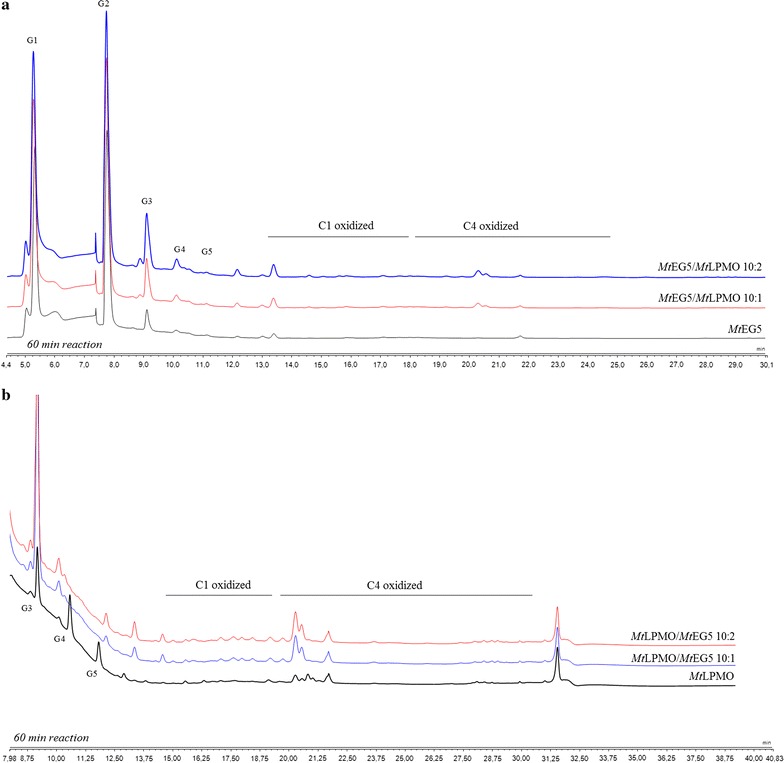
HPAEC-PAD chromatograms showing the synergistic effects of *Mt*EG5A and *Mt*LPMO9 when they are added together on 1.5% (w/v) PASC after 60 min of reaction. **a** Addition of *Mt*LPMO9 to *Mt*EG5A to a ratio EG5:LPMO 10:1 and 10:2 leads to increased formation of non-oxidized sugars. **b** Addition of *Mt*EG5A to *Mt*LPMO9 to a ratio LPMO:EG5 10:1 and 10:2 increases the release of oxidized sugars

**Fig. 9:**
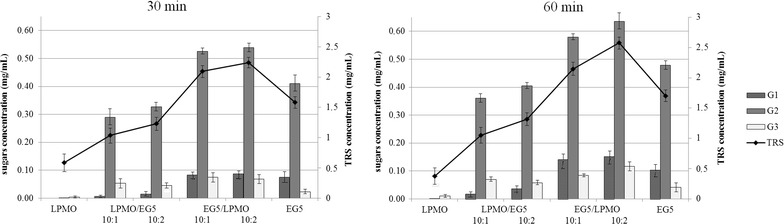
Determination of the soluble products from HPAEC analysis and the total neutral sugars (TRS) using the dinitrosalicylic acid method, after 30 and 60 min of PASC incubation with different combinations of *Mt*EG5A and *Mt*LPMO9

*Mt*EG5A was incubated with *Mt*LPMO9 at different enzyme combinations and enzyme loadings, at 50 °C, using PASC 1.5% (w/v), as shown in Table 1. Total neutral sugars and soluble products after 60 min of reaction are shown in Fig. 8. Data after 30 min of reaction are shown in Additional file 1: Figure S3. The corresponding chromatograms highlight the synergistic effects presented that refer to the release of both oxidized and non-oxidized oligosaccharides. The release of total non-oxidized sugars by *Mt*EG5A in the presence of *Mt*LPMO9, in a ratio of 10:1, was around 1.2 and 1.5 times higher at 30 and 60 min of the reaction, respectively, compared to the activity of pure *Mt*EG5A and pure *Mt*LPMO9 alone; it reached 1.3 and 1.6 times, respectively, when the EG:LPMO ratio was 10:2 (Fig. 9). Moreover, apart from the synergism in neutral sugars, there was synergism in release of oxidized sugars. Incubation of *Mt*EG5A with PASC resulted in hardly detectable oxidized gluco-oligosaccharides. In the presence of ascorbic acid, many oxidized gluco-oligosaccharides are formed by *Mt*LPMO9 when acting on PASC (within the first 60 min of the reaction, mainly C4 oxidation is detected). The combined addition of *Mt*EG5A and *Mt*LPMO9 in a ratio of 10:1 and in the presence of ascorbic acid resulted in a 1.4- and a 1.7-fold higher release of C1- and C4-oxidized products respectively, based on comparison of the sum of AUC (area under curve) determined by HPAEC, compared to *Mt*LPMO9 incubated with PASC only. When the LPMO: EG5 ratio was 10:2, the release rates of C1- and C4-oxidized products were 1.8- and 1.9-fold higher, respectively. After 60 min of reaction, the total release of oxidized sugars was approximately 2.5-fold greater than those released by *Mt*LPMO9 alone.

**Table 1:** Experimental combinations used for the assessment of combined activity of *Mt*EG5A and *Mt*LPMO9 for the hydrolysis of 1.5% (w/v) PASC

Reaction #	Enzyme combination	Relative proportion	Enzyme loading (mg/g substrate)
1	*Mt*EG5A		10
2	*Mt*EG5A/*Mt*LPMO9	10:1	11
3	*Mt*EG5A/*Mt*LPMO9	10:2	12
4	*Mt*LPMO9		10
5	*Mt*LPMO9/*Mt*EG5A	10:1	11
6	*Mt*LPMO9/*Mt*EG5A	10:2	12

**Fig. 10:**
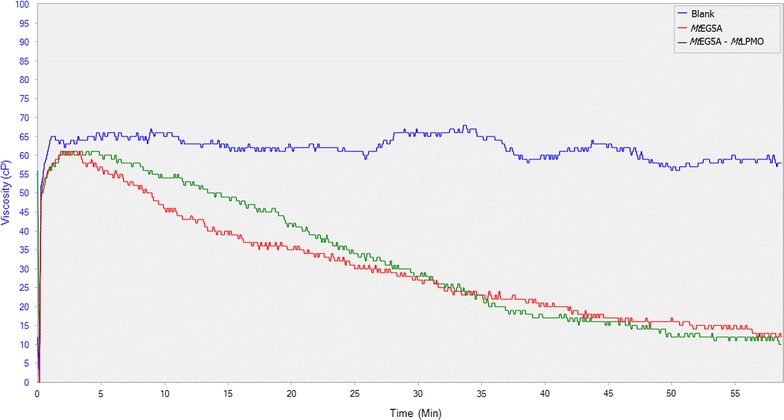
Dynamic viscosity experiments on 1.5% (w/v) PASC. *Mt*EG5A and *Mt*LPMO9 were added at a loading of 100 mg enzyme/g dry substrate. Mixing was kept at 50 rpm and viscosity reduction was monitored for 1 h

**Fig. 11:**
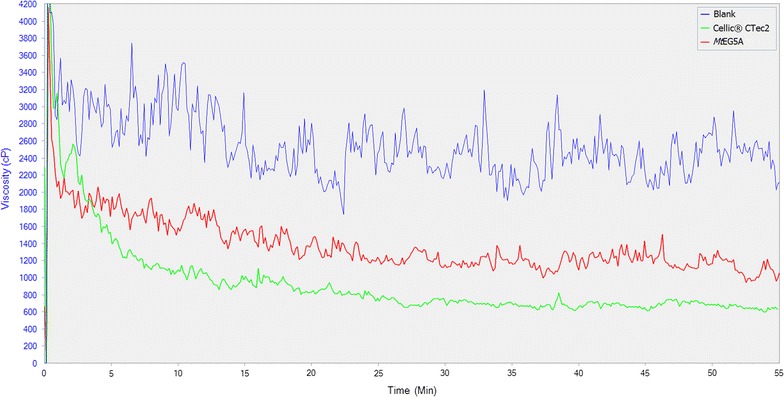
*Mt*EG5A activity on viscosity reduction of 10% w/w PWS. *Mt*EG5A was added at a loading of 100 mg enzyme/g dry substrate. Mixing was kept at 100 rpm and viscosity reduction was monitored for 1 h. Cellic^®^ CTec2 (Novozymes) was used to compare the endoglucanase of this study with a commercial cellulase preparation regarding the liquefaction of PWS

**Fig. 12:**
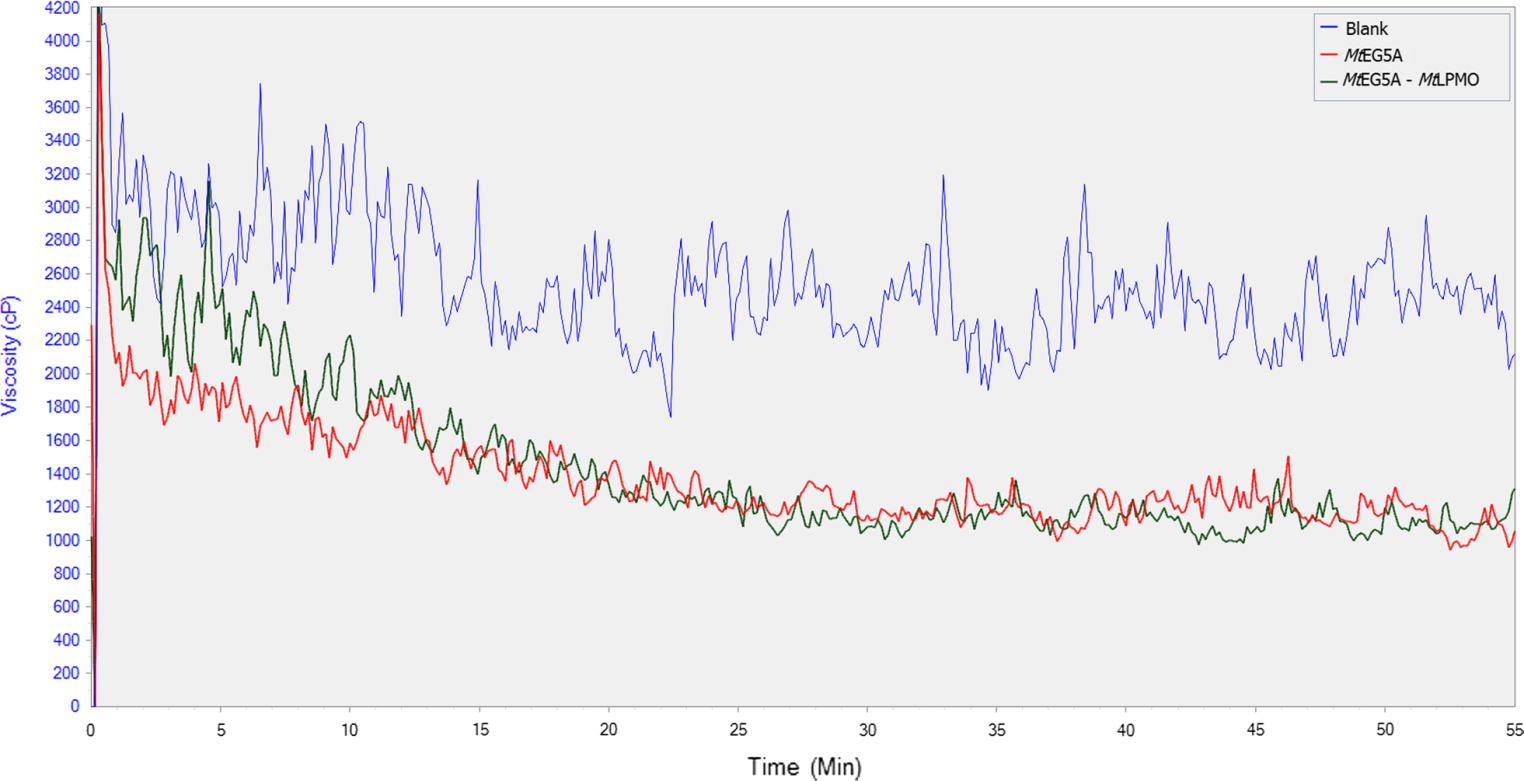
Assessment of combined activity of *Mt*EG5A and *Mt*LPMO9 on PWS liquefaction by simultaneous addition of the two enzymes in the reaction mixture. Both enzymes were added at a loading of 100 mg enzyme/g dry substrate. Mixing was kept at 100 rpm and viscosity reduction was monitored for 1 h

The effect of *Mt*EG5A, either alone or in combination with *Mt*LPMO9, on the liquefaction of PASC (Fig. 10) and PWS (Fig. 12) was also estimated via dynamic viscosity analysis experiments. *Mt*EG5A was shown to be able to reduce the viscosity of both PASC and PWS after incubation for 60 min. When compared to Cellic^®^ CTec2 (Novozymes), *Mt*EG5A did not reduce the viscosity as rapidly as the commercial enzyme mixture not only because the former is a processive EG that remains bound to the substrate until it dissociates it completely, but also because it is a monoenzyme, whereas Cellic^®^ CTec2 contains many different activities acting synergistically (Fig. 11). When *Mt*EG5A and *Mt*LPMO9 are added together, a less steep drop of viscosity was observed at the initial stage of the reaction (within the first 10 min) compared to *Mt*EG5A alone, whereas the difference in final viscosity was negligible (Fig. 10). The same results were obtained for PWS (Fig. 12).

## Discussion

*Mt*EG5A is an EG of GH family 5, consisting of an N-terminal CBM1 and a catalytic domain (CD). The gene encoding *Mt*EG5A from *M. thermophila* was heterologously expressed in *P. pastoris* under the regulation of the AOX1 promoter. After induction with methanol, the hydrolytic activity toward β-glucan and the accumulation of the recombinant enzyme in the culture broth increased significantly up to 53 U/mL after 6-day culture. Cultivation in bioreactor yielded 0.56 g/lt of pure *Mt*EG5A after 170 h of methanol induction. *P. pastoris* expression system has been successfully used for the heterologous expression of many GH family 5 EGs from various microorganisms, including *Volvariella volvacea* [61], *Phialophora* sp. G5. [62], *Penicillium echinulatum* [63] and *Penicillium decumbens* [64], *Gloeophyllum trabeum* [65], *A. niger* [66], *Aspergillus fumigatus* [67], and *Aspergillus nidulans* [68]. *Mt*EG5A has specific activity both on microcrystalline cellulose (Avicel) and β-glucan and exhibits properties, such as high temperature stability at 55 °C that render it a suitable candidate for use in biotechnological applications. Its relatively higher activity on microcrystalline cellulose in comparison with CMC is indicative of its processivity properties, which are further supported by the high ratio of soluble to insoluble products that are released by filter paper hydrolysis. *Mt*EG5A hydrolyzes the substrate by releasing soluble sugars as major products, while generating only a small proportion of new chain ends into the remaining, insoluble substrate [11, 69]. In contrast with *Mt*EG5A, the non-processive EGs yield typically 30–50% of insoluble reducing sugars when they cleave cellulosic substrates [45]. Opposing to other processive EGs that produce mainly G4 [1], processive EGs belonging to GH family 5 from *Saccharophagus degradans* [70], *Gloeophyllum trabeum* [11], and *Volvariella volvacea* [12] have been reported to release G2 as the main product. A less randomly acting endoglucanase from *Fusarium oxysporum* having higher preference toward glycosidic bonds near the end of cellulose chains has been reported to release mainly cellobiose from cello-oligosaccharides [71]. Their processivity does not require a CBM, so it has been proposed that it is attributed to the affinities of the glucose-binding subsites in the catalytic center, the same in chitinases, common ancestor [72]. Estimation of the substrate binding modes and processivity of the enzyme are also verified by the high G2/G3 ratios released from PASC hydrolysis. The high amount of released G2 compared to G3 has been reported previously in the literature for EGs of GH family 5 [73]. Comparison between *Mt*EG5A and *Mt*EG7A hydrolysis products on PASC revealed the differences in catalytic cleavage mode of these two enzymes. Quantitative analysis of the soluble hydrolysis products generated from PASC following incubation with *Mt*EG5A and *Mt*EG7A revealed different patterns, as a result of different enzyme binding and degradation patterns. The correlation of the enzyme specificity with the GH family has been studied by Vlasenko et al. [74], supporting that the structure–function relationship can be explained by the active site structure and the catalytic mechanism of the enzyme. GH family 7 EGs randomly cleave cellulose leading to the formation of various degradation products, while the activity of *Mt*EG5A appeared to be the release of G2 as the major end product from the cellulosic substrate. The ability of *Mt*EG5A to accumulate G2 as the main product from polysaccharide hydrolysis, as wells as from lignocellulosic materials (wheat straw, birch, and spruce), renders this enzyme a promising candidate for food technology applications related to the production of large amounts of non-digestible cello-oligosaccharides (COS). COS are resistant to digestion by human gastric and pancreatic enzymes, so they can be promising prebiotic candidates. The prebiotic potential of cellobiose has been investigated by in vitro fermentations in the presence of a human fecal inoculum, where G2 was found to increase the concentration of bifidobacteria and lactic acid bacteria, as well as the levels of short-chain fatty acids (SCFA) [75, 76]. Despite the fact that G2 is the main product of CBHs, the latter are very sensitive to G2 inhibition in contrast with EGs where G2 inhibition is much weaker [77], leading to the assumption that EGs could be widely used in applications targeting the efficient production of COS. Their possible use is further supported by their ability to liquefy rapidly and efficiently pretreated lignocellulosic materials such as wheat straw, under high dry matter loadings [18% (w/w) DM] [9].

When added together, *Mt*EG5A and *Mt*EG7A acted additively on PASC toward the release of glucose after 24 h of incubation. Endo–endo cooperative interactions between cellulases usually emerges from the fact that GH family 7 EGs serve to randomly hydrolytically cleave glycosidic linkages, whereas the processive GH family 5 enzymes rapidly cleave without dissociation [78]. *Mt*EG5A also showed a synergistic effect with *Mt*LPMO9 with an increased release of both oxidized and non-oxidized oligosaccharides, compared to the oligosaccharides release of each enzyme alone. The strong synergy of a LPMO9 enzyme with an EG I from *Trichoderma viride* in degrading amorphous cellulose has been reported in the literature [28], where the combined addition of the two enzymes resulted in a higher release of non-oxidized oligosaccharides, compared to the oligosaccharides release of each enzyme alone. In this study, there was given the first indication that synergy between an LPMO and an EG happens also on the level of formation of oxidative sugars, and this observation has not been reported before.

Dynamic viscosity analysis is a novel, sensitive, direct method for the measurement of the number of cleavages introduced in the polysaccharide substrate. This method is independent of the cleavage pattern of enzymes and oxidative regioselectivity [79], thus it can be used for monitoring and comparing the activity of EG and EG/LPMO combination on PASC and PWS. *Mt*EG5A was shown to be able to reduce the viscosity of both PASC and PWS. GH family 5 EGs have been reported in the literature to rapidly decrease the viscosity of CMC [12] as well as to reduce the viscosity of barley mash and improve the brewing process [80]. EG2 (Cel5a) has also been identified as a key component for the liquefaction of PWS [81]. When *Mt*EG5A and *Mt*LPMO9 are added together, the above discovered synergy regarding the release of neutral and oxidative sugars did not result in decrease of viscosity; the initial rate of reaction was lowered instead of increasing, thus suggesting that inhibition effects rather than synergy between the two enzymes occur. This could be explained as both *Mt*LPMO9 and *Mt*EG5A act competitively for the same binding sites, so *Mt*LPMO9 prevents *Mt*EG5A from binding on the substrate and making the initial cleavage or by the assumption that *Mt*LPMO9’s oxidative activity leads to some binding sites for EG5 being removed or rendered inaccessible. The substrate that was initially less accessible to *Mt*EG5A was degraded in a later phase of hydrolysis, thus reaching the same viscosity levels at the end of the reaction. Similar findings have also been reported by Eibinger et al. [31] regarding the combining effects of simultaneous addition of LPMO and CBHI on different cellulosic substrates.

## Conclusions

In the present study, the processive endoglucanase *Mt*EG5A from the thermophilic fungus *M. thermophila* was heterologously expressed in the methylotrophic yeast *P. pastoris* and the produced enzyme was purified and characterized. The enzyme featured high thermostability and was able to hydrolyze several natural substrates releasing cellobiose as the main product, features that reflect its potential use in different biotechnological applications. When its activity was tested together with a LPMO, there was a substantial increase in the formation of neutral and oxidative products. This is the first indication of synergy between LPMO and EG based on the yield of oxidative sugars.

## Additional file

**Additional file 1.** Additional figures and tables.

## Abbreviations

EG: endoglucanase
LPMO: lytic polysaccharide monooxygenase
PASC: phosphoric acid swollen cellulose
CMC: carboxymethyl cellulose
PWS: pretreated wheat straw
