# Supplementary material for: Recombinant expression of thermostable processive MtEG5 endoglucanase and its synergism with MtLPMO from Myceliophthora thermophila during the hydrolysis of lignocellulosic substrates
Source: Biotechnol Biofuels. 2017 May 15;10:126. doi: 10.1186/s13068-017-0813-1 (PMC5432998; doi:10.1186/s13068-017-0813-1)

**Recombinant expression of thermostable processive *Mt*EG5 endoglucanase and its synergism with *Mt*LPMO from *Myceliophthora thermophila***

**Supplementary Material**

**TABLES**

**Table 1.** Primers (**A**) and conditions (**B**) used for the amplification of *eg5a* gene through PCR (#1), removal of introns (#2-4) and final overlapping PCR (#5). Colored sequences represent the restriction sites of *ClaI* (*red*) and *XbaI* enzymes (*purple*), as well as the complementary DNA fragments that allowed the hybridization reaction and the amplification of the generated “*fusion*” fragment by overlapping PCR (*blue*: annealing, *green*: overhang).

**Table S**1A

|  | primer sequence | |
| --- | --- | --- |
| *St*EG5a86753**F** (32 bp) | 5' GC**A TCG AT**G CAA AGT GGT TGG GAG CAA TG 3' | |
|  | *ClaI* | |
| *St*EG5a86753**R** (32 bp) | 5' CG**T CTA GA**G GCA AGT ACT TCT TCA AGA TCG AG 3' | |
|  | *XbaI* | |
|  | | primer sequence (OE-PCR) |
| *St*EG5a86753**e1R** (37 bp) | | 5' **CCA TCA TTG ATG AGC GTC TGA ATC GCC GAA GTC GAC G** 3' |
| *St*EG5a86753**e2F**  (24 bp) | | 5' ACG CTC ATC AAT GAT GGA TAC AAC 3' |
| *St*EG5a86753**e2R** (39 bp) | | 5' **GTG CCC GAA GGA GGC TCG AAC GAG TAC ATG TAG TCG CCC** 3' |
| *St*EG5a86753**e3F** (19 bp) | | 5' AGC CTC CTT CGG GCA CCG G 3' |

**Table S**1B

| PCR | Primers | Target Fragment | Conditions |
| --- | --- | --- | --- |
| **#1** | **EF - ER** | 1570 bp | annealing: 56^o^C/10s extension: 70^o^C/25s, 30 cycles |
| **#2** | **EF - Ee1R** | 315 bp | annealing: 56^o^C/10s extension: 70^o^C/4s, 30 cycles |
| **#3** | **Ee2F - Ee2R** | 745 bp | annealing: 56^o^C/10s extension: 56^o^C/12s, 30 cycles |
| **#4** | **Ee3F - ER** | 1600 bp | annealing: 56^o^C/10s extension: 70^o^C/1s, 30 cycles |
| **#5** | **EF - ER** | 1122 bp | annealing: 56^o^C/10s extension: 59^o^C/26s, 45 cycles |

**Table S2.** Properties of *Mt*EG5A obtained from genome analysis.

| Genome Portal ID | 86753 |
| --- | --- |
| Chromosome | 1: 2823610 - 2825549 |
| Family | Glycoside hydrolase 5 |
| Domains | CBM1, [Pfam: PR00734, InterProScan] |
| Gene (translation) | 1170 bp |
| Gene (trancription) [3’UTP, 5’UTP] | 1940 bp |
| Protein | 389 aa |
| Exons | 3 |
| Secretion signal | MKSSILASVFATGAVA (17 aa) |
| Theoretical predicted MW | 40.85 kDa |
| theoretical pI | 5.07 |
| Glycosylation sites N-Glyc | 3 |
| Glycosylation sites O-Glyc | 17 |

**Table S3.** Primers (**A**) and conditions (**B**) used for the amplification of *lpmo9* gene. Colored sequences represent the restriction sites of *BstBI* (*red*) and *XbaI* enzymes (*purple*).

| *Mt*LPMO9_46583**F** (30 bp) | 5' GC **T TCG AA** A TGT CCA AGG CCT CTG CTC TCC 3' |
| --- | --- |
|  | *BstBI* |
| *Mt*LPMO9_46583**R** (29 bp) | 5' CG**T CTA GA**C ACT GGG AGT ACC ACT CGT TG 3' |
|  | *XbaI* |

**FIGURES**

**Figure S1.** X-ray diffraction pattern of PASC generated with 85% phosphoric acid. The reflection of face around 2*θ* =20° is attributed to amorphous parts, while the reflection centered on 2*θ* =21° represents the crystalline region. Diffraction spectrum of Avicel PH-101 was used as a reference sample for crystalline cellulose.


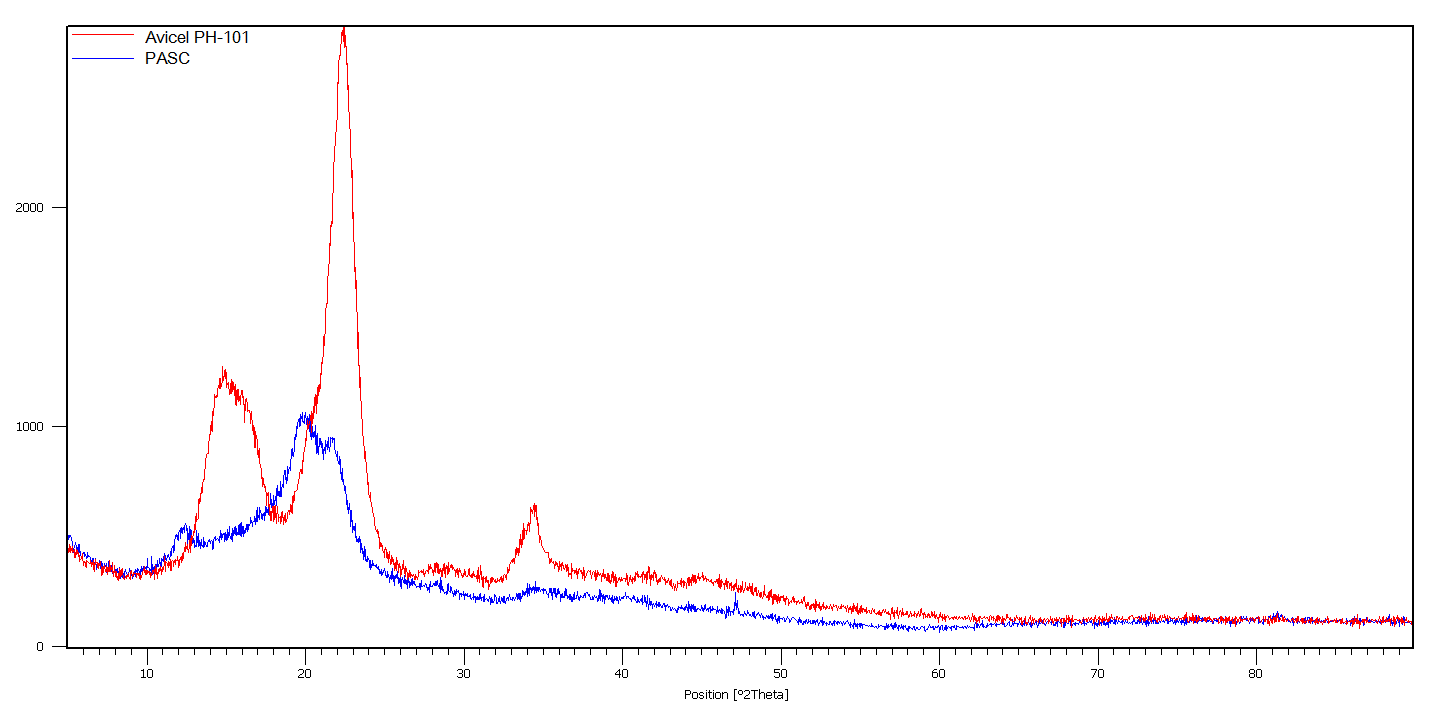


**Figure S2.** HPAEC-PAD chromatograms showing the product profile of *Mt*EG5A and *Mt*EG7A activity on amorphous cellulose PASC after incubation for 24 hours at 50 ^ο^C.

**
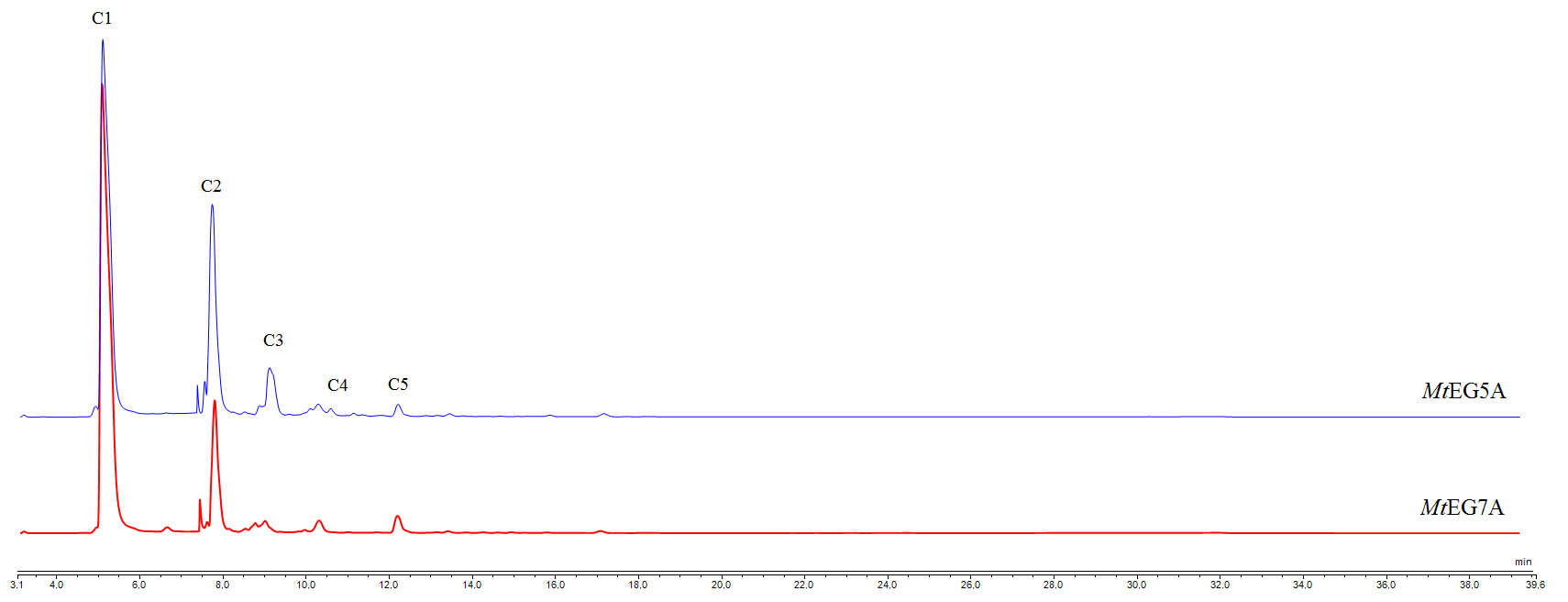
**

**Figure S3**. HPAEC-PAD chromatograms showing the synergistic effects of *Mt*EG5A and *Mt*LPMO9 when they are added together on 1.5% (w/v) PASC after 30 min of reaction. (**A**) Addition of *Mt*LPMO9 to *Mt*EG5A to a ratio EG5:LPMO 10:1 and 10:2 leads to increased formation of non-oxidized sugars. (**B**) Addition of *Mt*EG5A to *Mt*LPMO9 to a ratio LPMO:EG5 10:1 and 10:2 increases the release of oxidized sugars.


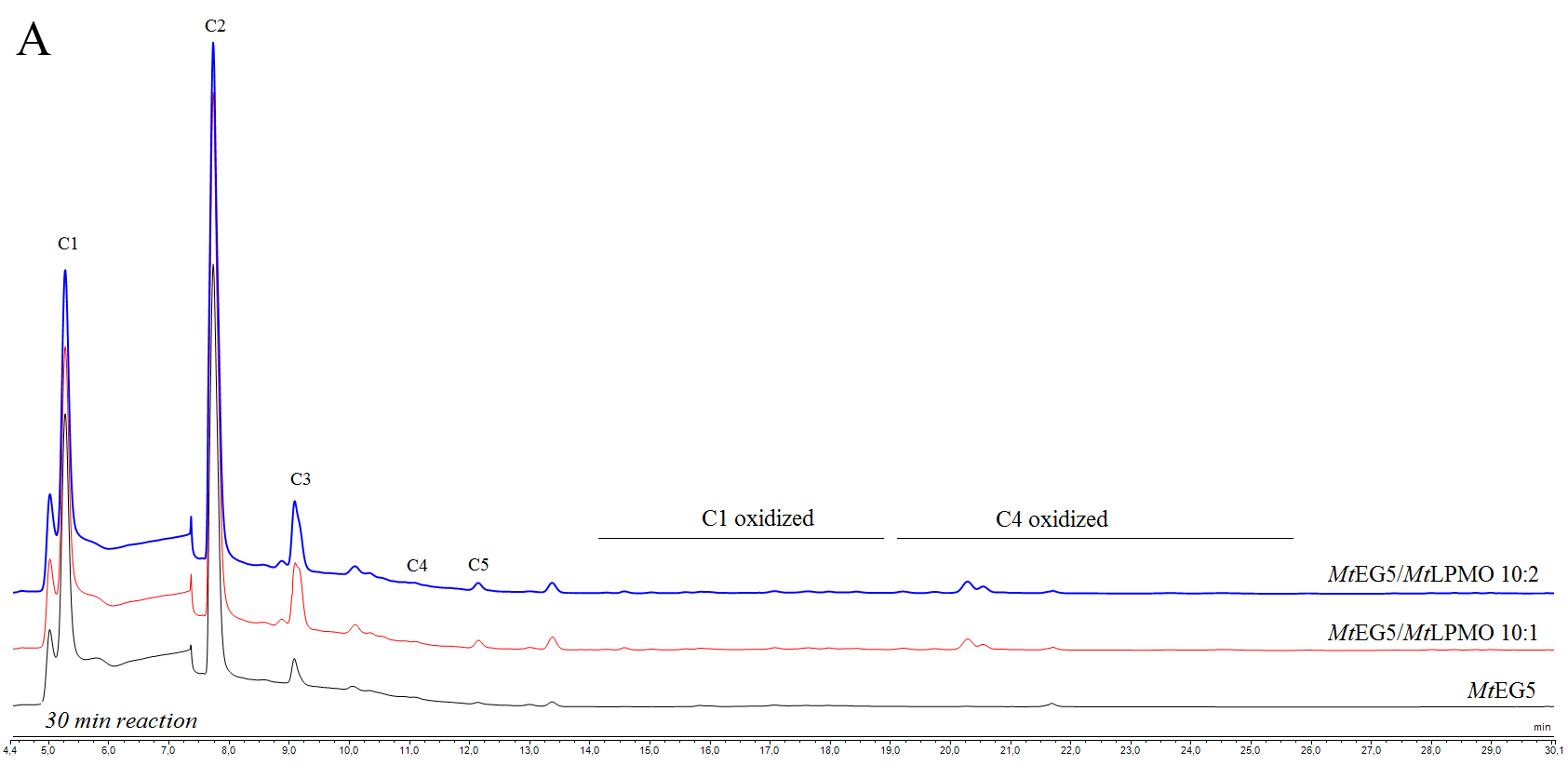


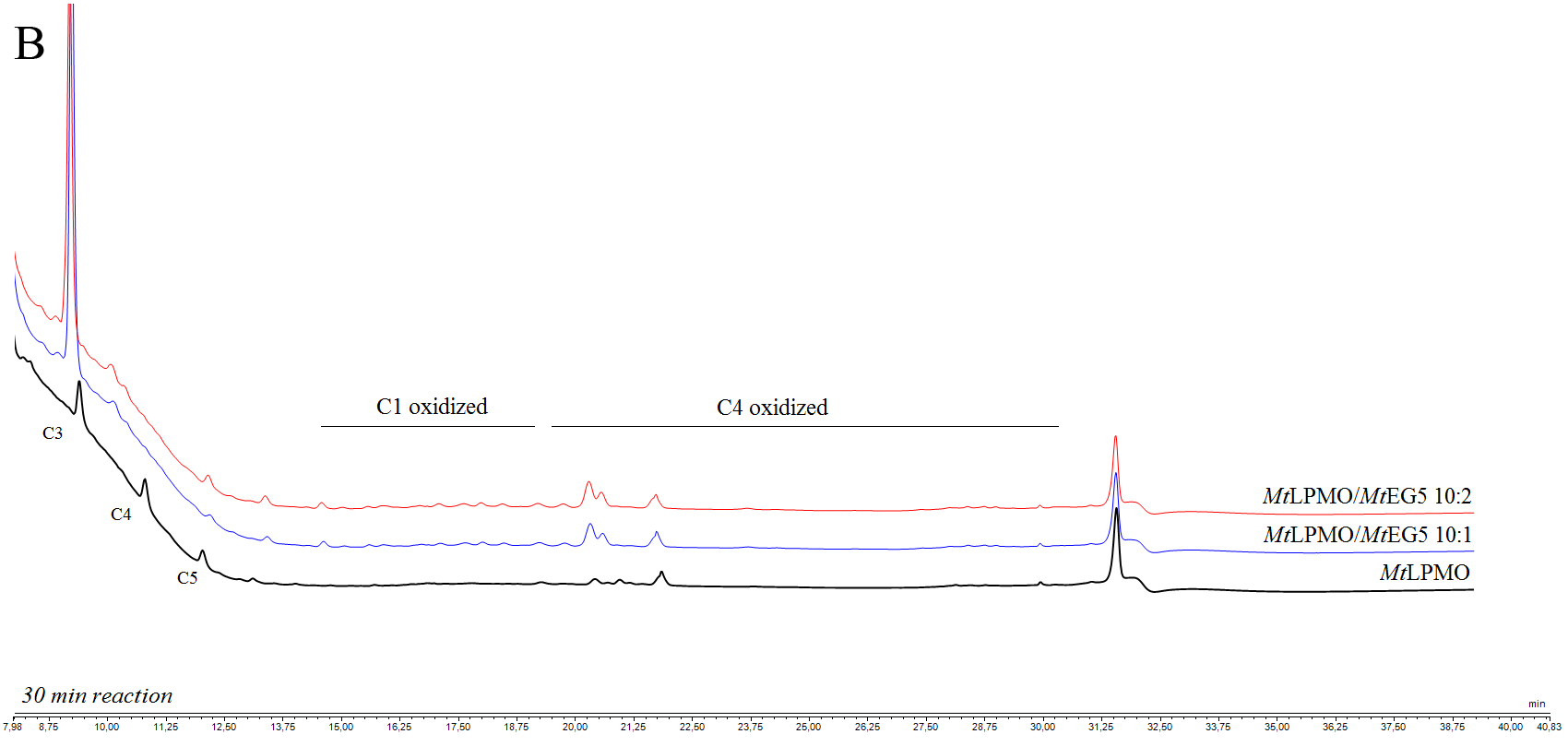

Supplement: Supplementary file 1 — Additional file 1. Additional figures and tables. [file 13068_2017_813_MOESM1_ESM.doc]
